# Effects of maize straw treated with various levels of CaO and moisture on composition, structure, and digestion by *in vitro* gas production

**DOI:** 10.5713/ab.21.0184

**Published:** 2021-08-25

**Authors:** Mingjun Shi, Zhanxia Ma, Yujia Tian, Xuewei Zhang, Huiyong Shan

**Affiliations:** 1 Tianjin Key Laboratory of Agricultural Animal Breeding and Healthy Husbandry, College of Animal Science and Veterinary Medicine, Tianjin Agricultural University, Tianjin 300384, China; 2 College of Engineering and Technology, Tianjin Agricultural University, Tianjin 300392, China

**Keywords:** Maize Straw, X-ray Fluorescence, Scanning Electron Microscopes, *In Vitro*

## Abstract

**Objective:**

The objective of this study was to explore the effects of maize straw treated with calcium oxide (CaO) and various moisture, on the composition and molecular structure of the fiber, and gas production by fermentation in an *in vitro* rumen environment.

**Methods:**

The experiment used 4×3 Factorial treatment. Maize straws were treated with 4 concentrations of CaO (0%, 3%, 5%, and 7% of dry straw weight) and 3 moisture contents (40%, 50%, and 60%). Scanning electron microscopy, Fourier transform infrared spectroscopy and X-ray fluorescence spectroscopy were employed to measure the surface texture, secondary molecular structure of carbohydrate, and calcium (Ca) content of the maize straw, respectively. The correlation of secondary molecular structures and fiber components of maize straw were analyzed by CORR procedure of SAS 9.2. *In vitro* rumen fermentation was performed for 6, 12, 24, 48, and 72 h to measure gas production.

**Results:**

Overall, the moisture factor had no obvious effect on the experimental results. Neutral detergent fiber (NDF), acid detergent fiber, acid detergent lignin, hemicellulose and cellulose contents decreased (p<0.05) with increasing concentrations of CaO treatment. Surface and secondary molecular structure of maize straw were affected by various CaO and moisture treatments. NDF had positive correlation (p<0.01) with Cell-H (H, height), Cell-A (A, area), CHO-2-H. Hemicellulose had positive correlation (p<0.01) with Lignin-H, Lignin-A, Cell-H, Cell-A. Ca content of maize straw increased as the concentration of CaO was increased (p<0.01). Gas production was highest in the group treated with 7% CaO.

**Conclusion:**

CaO can adhere to the surface of the maize straw, and then improve the digestibility of the maize straw in ruminants by modifying the structure of lignocellulose and facilitating the maize straw for microbial degradation.

## INTRODUCTION

Maize straw is the maize plant left after removing maize grains and is one of the most abundant biomass energy resources. Maize straws are widely distributed and cheap. At present, in China, a fraction of maize straw is used in manufacturing paper, board and other industrial raw materials while most of the maize straw is burned or thrown away, resulting in significant waste of natural resources. Discarding the straw by burning and dumping in the field does not only affect the air quality critically, but also aggravates the greenhouse gas emission [1].

Maize straw is considered as an alternative forage source in feeding ruminants when other forage supplies are limited. However, the maize straw has high cellulose and lignin content, and the lignin and crystalline cellulose are connected by covalent bonds to form three-dimensional network structure. This structure reduces the digestibility and palatability of the maize straw [2]. Therefore, maize straw is hardly used in feeding to ruminants. Several studies explored various means to modify the three-dimensional network structure in order that maize straw could be efficiently utilized by ruminants. There are many methods including physical, chemical, and biological methods to pretreat maize straw to improve the digestibility and palatability [3]. Physical means of intervention can ameliorate the palatability of maize straw and improve its nutritional value. But most of the shortening process to improve digestibility needs special equipment requiring high energy input and with low efficiency, which greatly limit the use of the physical process in the production and application. Biological methods use microorganisms and enzymes to treat maize stalk, and those methods consume low energy and mild reaction conditions [2,4]. However, only a few types of microorganisms can effectively degrade lignin, and further elucidation is necessary to screen the lignin-degrading microorganisms. In chemical treatment, maize straw is treated with acid or alkali. Alkali treatment, at an optimum concentration breaks the ester bond between the structural molecules of lignocellulose resulting in more gaps in its structure and increases the surface area of the internal structure. These changes produce a change in the population of rumen microorganisms [5]. Alkali treatment reduced the content of neutral detergent fiber (NDF), and the ratio of NDF/organic matter in rice straw [6]. In recent years, several investigations have been using calcium oxide (CaO) to treat maize straw to explore its effect on digestion and degradation in ruminants. CaO, commonly known as quicklime, is a chemical compound-that has plenty of natural sources. Its production can be mechanized on a large scale with low production cost. Consequently, CaO can be used to treat maize straw to increase its nutritive value. Some studies have analyzed the molecular structure of canola [7], barley [8], dried distillers grains with solubles [9], but the change of molecular structure of maize straw treated with CaO has not been reported. Further studies are needed to elucidate the ultra-structural changes of the maize straw upon CaO treatment and the mechanism behind the structural modification.

The development of infrared spectroscopy enables the identification of organic, inorganic and polymer materials today. Such analytical technology uses infrared radiation that passes through the substance to be analyzed. When radiation is absorbed by or transmitted from the substance, resultant spectrum represents molecular fingerprint of the sample. This resultant image elucidates its structural characteristics [7]. Fourier transform infrared spectroscopy (FTIR) provides quantitative analysis of complex substances and information about surface and interfacial appearances. The scanning electron microscope (SEM) amplifies and restores the surface morphology of the sample by collecting the secondary electron signal generated from the interaction of the electron beam [10]. X-ray fluorescence spectrometry (XRF) uses primary X-rays to excite the atoms in the substance to be tested. The excited atom produces fluorescence and, the composition of the substance is analyzed precisely and rapidly. It is widely used to determine almost all the elements in soil, crude oil, and atmosphere in the metallurgical industry.

Previous researchers mainly considered the digestion of straw treated with CaO in animals by using *in vivo* and *in vitro* methods [11], but few conducted systematic and in-depth studies on the structural changes of straw after treatment with CaO. In this study, FTIR, SEM, and XRF methods were adopted to comprehensively analyze the structural changes of maize straw after treatment with CaO to fully understand the metabolic mechanism of structure change of the treated maize straw. We hypothesize that CaO treatment could affect the fiber composition, molecular structure, and surface topography of maize straw probably due to adhesion property of CaO to maize straw treated with various moisture contents. Fiber composition, molecular structure, electron microscopic structure and CaO adhesion to straw were determined. In addition, gas production in an *in vitro* rumen environment was explored to determine the ability of CaO to modify the internal structure of maize straw and improve its degradation potential in the rumen. The outcome of the study will provide guidance for rational use of maize straw resources.

## MATERIALS AND METHODS

### Animals and diet

*In vitro* test was carried out in the key laboratory of College of Animal Science, Anhui Science and Technology University (Feng yang, China). Rumen fluid was collected from four non-pregnant Holstein cows (610±20 kg) that fitted with rumen fistulas. Total mixed ration was fed to dairy cattle at 06:00 am and 6:00 pm respectively according to Nation Research Council (NRC) 1.3 maintenance nutrients levels (Table 1). Water was attainable optionally. This animal experiment was approved by the Ethics Committee on animals of Tianjin Agricultural University (2020LLSC12).

### Material source and processing

Five plots of one square meter area of land situated in Hebei Province of China were randomly chosen for collecting samples of maize straw (Maize variety: Yufeng 303). All the maize straws were cut into 5 to 8 cm length pieces. This experiment used 4×3 factorial treatment with 5 replications. Sixty 1.5 kg samples were treated with CaO at four different concentrations (0%, 3%, 5%, and 7% of dry straw weight) and three moisture contents (40%, 50%, and 60%). Each treatment was sprayed with slurry, which was made of CaO suspended in water, and mixed evenly. All samples were placed in 1-L fermentation tank under anaerobic condition for 30 d. Following fermentation, the samples were dried at 65°C in an air oven for 48 h, and subsequently kept in room temperature for another 48 h to complete the process of air drying. All samples were stored in plastic bags until further analysis.

### Analysis of fiber composition

All straw samples were ground and filtered through a sieve with a pore size of 0.425 mm then put into the filter bags (F58, ANKOM Technology, Macedon, NY, USA). The NDF, acid detergent fiber (ADF), and acid detergent lignin (ADL) were analyzed using an ANKOM A2000 fiber analyzer (ANKOM Technology, USA). The contents of NDF, ADF, ADL were determined using a previously described method in Van Soest et al [12]. The contents of hemicellulose and cellulose were calculated using the following formula:

Hemicellulose=NDF-ADF;Cellulose=ADF-ADL.

### Scanning electron microscopic analysis

The surface structure topography of the maize straw was analyzed using S-4800 SEM (Hitachi, Ltd. Tokyo, Japan). Samples were sputter-coated with gold foil and observed at 10× magnification using 3 kV electron beam [13].

### Fourier transform infrared spectroscopic analysis

Maize straw samples were ground and filtered through a sieve with a pore size of 0.25 mm. Spectral features of carbohydrates in the samples were measured using FTIR-7600 (Lambda Scientific, Adelaide, Australia) in the molecular spectroscopy laboratory at Tianjin Agricultural University. Standard laboratory procedures were followed. Spectra were collected on a transmission mode at the mid-IR (4,000 to 800 cm^−1^) area of the electromagnetic field with a spectral resolution of 4 cm^−1^. Sixty-four scans were co-added, and five replicates per sample was performed [14]. Due to CaO and water vapor in the air, it was necessary to perform a blank reading before the spectral collection of each sample. Data analysis about functional groups of carbohydrate in the maize straw was performed using OMNIC 8.2 software (Thermo Nicolet Corporation, Madison, WI, USA).

### X-ray fluorescence spectroscopic analysis

Ca contents in the maize straw samples were determined using ZSX Primus XRF (Rigaku, Beijing, China). The samples were placed in a tightly closed chamber. The samples were shaped into discs with a thickness of 1 cm and a diameter of 3 cm using a tablet press. The XRF was equipped with the FP-Multi software (Panalytical, Almelo, Netherlands). The samples were loaded, and the measurements were obtained following the instructions of the FP-Multi software [15].

### *In vitro* rumen fermentation and gas production

We followed the method of *in vitro* fermentation proposed by Menke et al [16] and Ren et al [17] to determine the gas production of maize straw. Samples were treated with different concentrations of CaO and different moisture contents in an *in vitro* rumen environment. Approximately 0.5 g maize straw was used as fermentation substrate and the straw was mixed with 50 mL buffer solution and 25 mL rumen liquid at 39°C in a fermentation bottle. The CO_2_ was continuously added to the mixture and stirred to create an anerobic environment. The gas production was measured in the *in vitro* fermentation bottle (to simulate rumen) by estimating the pressure using JYB pressure level sensor (Guangkong, Guangzhou, China). The pressure was monitored at 6, 12, 24, 48, and 72 h during the process of fermentation. The formula to calculate the production of gas was as follows: GP (mL) = P (MPa)×5.7512–0.1591 (GP, gas production; P, gas pressure).

### Statistical analyses

Fiber composition, CaO content, fiber secondary molecular structure and rumen simulated *in vitro* gas production were statistically analyzed using MIXED models procedures in the statistical software SAS (version 9.2, SAS Institute Inc., Cary, NC, USA). The model used in the analysis was Y_ij_ = μ+F_i_+F_j_+F_ij_+e_ij_. Y_ij_ is the dependent variable, μ is the average value of variables i and j, F_i_ is the difference of CaO concentrations which is a fixed factor, and F_j_ is the difference of moisture contents which is another fixed factor, F_ij_ is the fixed interaction between the CaO treatment and moisture, and e_ij_ is the random error related to variables i and j.

### Correlation analysis

The relationship between secondary molecular structures and fiber components of the maize straw in different treatment groups was analyzed using the CORR procedure of SAS 9.2 (SAS Institute Inc., USA) [18]. In this study, significant difference was considered at p<0.05 and p<0.1 for the mixed model and correlation analysis, respectively. The comparison between different treatments was carried out using Tukey-Kramer method, and a SAS macro called “pdmix800.sas” (SAS Institute Inc., USA) [18] was used to denote the letter for each treatment mean at the significance level of 0.05.

## RESULTS

### Effects of different concentrations of CaO and various moisture contents on fiber composition of maize straw

The NDF, ADF, ADL, hemicellulose, and cellulose contents are shown in Table 2. CaO concentration affected the NDF, ADF, ADL, hemicellulose (p<0.01) and cellulose (p<0.05) contents in various treatment groups of the maize straw. However, moisture content and its interaction with CaO concentration did not affect them. As CaO concentration increased from 0% to 7%, NDF percent decreased from 76.28% to 64.97%, ADF decreased from 48.67% to 45.68%, ADL and hemicellulose decreased from 5.20% to 4.24%, 27.43% to 18.87%, respectively.

### Effects of different concentrations of CaO and various moisture contents on the fiber structure of maize straw

#### Scanning electron microscopic analysis of surface structure of maize straw

**Figure 1:**
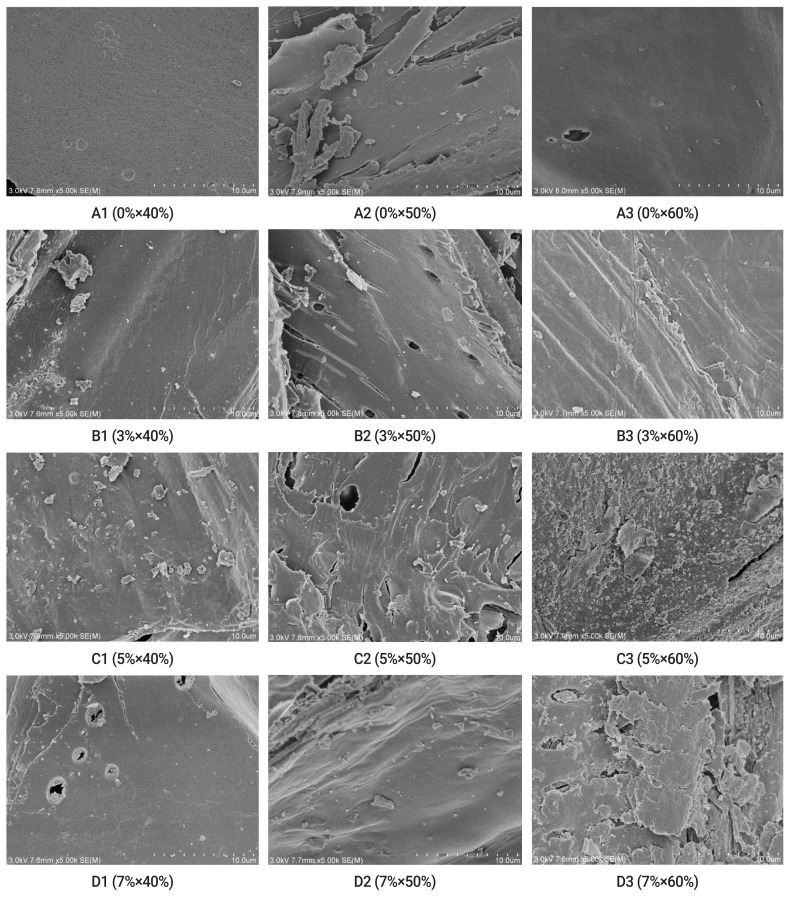
Scanning electron microscopic images of maize straw surface morphology in different treatment groups. Maize straws were treated with 4 concentrations of CaO (0%, 3%, 5%, and 7% of dry straw weight) and 3 moisture contents (40%, 50%, and 60%), the pictures show that with the increase of CaO concentration, the structural integrity of maize straws were damaged to varying degrees.

The images of surface morphology in various treatment groups of the maize straw are shown in Figure 1. The SEM images show that: the structural integrity of maize straw was damaged with the increasing concentrations of CaO. In the treatment group 0%×40% (Figure A1), the surface of straw was smooth and tightly wrapped by wax, and the structure appeared to be normal. In the group 3%×40% (Figure B1), the surface structure of straw was normal and healthy, there was no collapsing phenomenon, only a small amount of debris was seen, and there was no change in the long tube structure of straws. In the group 5%×50% (Figure C2) and 5%×60% (Figure C3), the straw surface was severely damaged, the long tube structure was torn, there were uneven folds and fragments and disordered holes on the surface of straws were seen. In the group 7%×60% (Figure D3), the straw surface was damaged to a greater degree, collapsing phenomenon was obvious and severe, and there were distinct flaky structures and many holes. These surface structure injuries lead to damage of the internal molecular structures.

#### Molecular structure carbohydrate features in different combinations of maize straw

Secondary molecular structures of carbohydrate from different treatment groups of maize straw are presented in Table 3. The functional groups analyzed in this study were lignin, structure of carbohydrate (STCHO), cellulose compound (Cell) and total carbohydrates (CHO). CaO had effects on Lignin-H, Cell-H, Cell-A, CHO-1-A (p< 0.01) and Lignin-A, CHO-1-H, CHO-2-H, CHO-3-A (p<0.05). Compared with 0% CaO group, the peak height and peak area of functional groups in 3%, 5%, and 7% groups tended to decrease. Among them, 5% CaO group had the lowest value in Lignin-H (2.67×10^−3^ vs 1.45×10^−3^), Lignin-A (69.37×10^−3^ vs 29.23×10^−3^), CHO-1-H (6.56×10^−3^ vs 4.91×10^−3^), CHO-1-A (137.10×10^−3^ vs 100.50×10^−3^), CHO-2-H (5.17×10^−3^ vs 3.43×10^−3^), CHO-3-A (3,184.10×10^−3^ vs 2,347.00×10^−3^). And 7% CaO group had the lowest value in Cell-H (8.50×10^−3^ vs 2.36×10^−3^) and Cell-A (413.70×10^−3^ vs 120.70×10^−3^). Maize straws treated with moisture affected Cell-H, Cell-A, CHO-1-A. There was no significant effect on secondary molecular structures of carbohydrate between CaO and moisture interaction.

#### Correlation analysis between secondary molecular structures and fiber components of maize straw among different treatment groups

The results of the correlation analysis are presented in Table 4. The NDF had positive correlation with Cell-H, Cell-A, CHO-2-H (p<0.01), and Lignin-H, CHO-1-H, CHO-1-A, CHO-2-A, CHO-3-A, CHO-3-H (p<0.05). The hemicellulose from different treatment groups had a positive correlation with Lignin-H, Lignin-A, Cell-H, Cell-A (p<0.01), and CHO-1-H, CHO-1-A, CHO-2-H, CHO-3-H, CHO-3-A (p<0.05). In addition, cellulose had positive correlation with CHO-2-H (p<0.01), and Cell-H, Cell-A, CHO-2-A, CHO-3-H (p<0.05).

#### X-ray fluorescence analysis of content of Ca in maize straws

The contents of CaO and Ca are provided in Table 5, and they were different among various treatment groups. The study found that the contents of CaO and Ca were increased in the maize straws with increasing concentrations of CaO treatments (p<0.01). The contents of CaO for 7% and 5% treatments were much higher than those for 0% treatment (p<0.01), and 3% treatment (p<0.05). The statistical significance for the contents of Ca, was like that for CaO. The moisture contents of maize straws had no statistically significant effects on the content of CaO and Ca, but the interaction between CaO and moisture content had effect on the contents of CaO and Ca (p<0.01). In general, the Ca and CaO contents of treatments with 7% CaO were significantly higher than those with all other groups.

### Gas production in an *in vitro* simulated rumen environment from the maize straws treated with CaO and moisture

The results of gas production at 6, 12, 24, 48, and 72 h under fermentation conditions are shown in Table 6. CaO affects the gas production of maize straw (p<0.01), while the effect of moisture and these two variables (CaO×moisture) on *in vitro* gas production was not statistically significant. Among them, 7% group had higher gas production at 6, 12, 24, 48, and 72 h than 0%, 3%, and 5% treatment groups. With the increasing of CaO treatment concentrations, gas production increased from 5.70 mL to 8.44 mL at 6 h of *in vitro* fermentation. At 12 h of *in vitro* fermentation, gas production increased from 29.11 mL to 35.10 mL. In 24, 48, 72 h, gas production increased from 46.43 mL to 51.26 mL, 55.18 mL to 61.12 mL, 66.21 mL to 73.99 mL, respectively. And after 48 h, the gas production in 5% and 7% treatment groups was increased gradually. Therefore, adding CaO to maize straw can considerably increase the gas production *in vitro*, that increases with the increase of CaO.

## DISCUSSION

### Effects of different concentration of CaO and various moisture contents on fiber composition of maize straw

With the increasing levels of CaO treatment, NDF, ADF, ADL, hemicellulose, and cellulose contents of maize straw declined. There is evidence that alkali agents can saponify the uronic acid chains attached to the xylan backbone to produce, charged carboxyl groups, and split the chains of lignin, hemicellulose, and others [3]. Further, hemicellulose of straw can be solubilized by alkaline treatment and consequently the alkaline treatment results in reduction of NDF contents [11]. Adding CaO to Rhodes grass at rate of 40 g/kg dry matter, significantly altered the NDF and ADF contents and CaO levels had a negative linear correlation with the contents of NDF and ADF [19]. These outcomes are consistent with the results of the current study. In the current study, there was no significant influence of the moisture contents and the interaction of CaO and moisture on fiber composition. The effectiveness of CaO treatment on fiber contents is obvious from the current and other studies. However, the mechanism behind this phenomenon is unknown and in-depth analysis of the molecular structure of the maize straw fiber could shed light on understanding of this mechanism.

### Evaluation of fiber structure of the maize straws treated with different combinations of CaO and moisture

Scanning electron microscopic analysis of surface structure of the maize straw: Maize straw contains an abundant amount of lignocellulose which is composed of cellulose, hemicellulose, and lignin [20]. Lignin is found between cellulose and hemicellulose and connected with carbohydrate polymers through hydrogen and covalent bonds, this morphology prevents the degradation of lignin. When the structure of the fiber is damaged, the lignin and part of the cellulose are disoriented and lost. Consequently, the surface of straw becomes rough, collapsed and fragmented [21]. Highly efficient bacterial strains from humus were used to degrade maize straw [1]. By increasing the time allowed for degradation, the waxy silicified layer of maize straw gradually thinned and disappeared. Subsequently, the lower epidermis was completely exposed. In the current study, CaO was used to degrade the maize straw, and a similar phenomenon was observed. In contrast, the surface of the untreated maize straw appeared to be smooth, tightly wrapped by wax, and healthy. Upon CaO treatment, the surface layer of straw became injured and this damage was obvious in the groups of 5%×50%, 5%×60%, and 7%×60% (CaO×moisture). The waxy part was lost, the surface area of outer wall was increased, the cell wall was stretched, the pores became dilated, the long tube structure of the maize straw was completely damaged, and the fibers were mostly fragmented. Therefore, CaO treatment can effectively degrade the network structure of cellulose and lignin, and thus improve the nutrient composition, digestibility, and utilization rate of the maize straw [11].

#### Evaluation of secondary molecular structure using Fourier infrared spectroscopy and correlation analysis between secondary molecular structure and fiber components of the maize straw treated differently

To evaluate the quality of carbohydrates in feedstuffs, it is necessary to analyze their molecular structure in addition to their components. Changes in the molecular structure of protein and carbohydrate can affect the nutritional value, digestibility, and utilization of feed. FTIR can detect the molecular structure of complex plants. In this detection process, the absorption peak of carbohydrates caused by the tensile vibration of C-O, C-C, and C-O-H bonds is measured [8]. The absorption values of carbohydrate functional groups in various treatment groups should differ in the present study and therefore the corresponding internal molecular structures appear different. The peak value or peak area of the corresponding structural components can be used as a parameter to predict the nutritional value of feed materials [22]. Previous studies found that the change of molecular structure affects the composition of nutrients in the feed and rumen digestibility [14].

In the current study, the absorption values of carbohydrate functional groups in each treatment group were different, which may diversely influence the process of degradation in the rumen. The current study found that ADF, NDF, ADL, hemicellulose and cellulose contents in the maize straw gradually decreased with increasing concentration of CaO treatments. Among them, NDF and hemicellulose had a positive correlation with the peak height of lignin, and strong positive correlations with the peak area and peak height of cellulose complex. In contrast, ADF and ADL had poor correlations with carbohydrate. In varying proportions of hulless barley and pure wheat DDGS mixture, NDF, hemicellulose and cellulose had strong correlations with the spectral intensities of lignin and other cell compounds [23]. Present result is comparable with this previous study. But another study [9] recognized that ADF was negatively correlated with most of the carbohydrate peak areas, such as STCHO, cell compounds, CHO-1, and CHO-2 in the mixtures of maize and barley DDGS. In that study, ADF, NDF, and ADL were positively correlated with the peak area of STCHO and CHO. This result is inconsistent with the results of the present study and the variation may be due to the different feed types and different processing technologies employed. Therefore, further investigations on the molecular structures and nutrient metabolism of different feed types are critically important.

#### Analysis of the contents of CaO and Ca in the treated maize straw using X-ray fluorescence microscopy

XRF spectrometry is a technology by which qualities and quantities of elements in the test samples can be analyzed [24]. The primary X-ray photons or other microscopic particles are used to excite the atoms in the matter to be measured. When these molecules are excited, they produce secondary X-rays. The characteristics of emitted rays are used to study the substances to be analyzed.

Some studies explored the accumulation of nano titanium dioxide by duckweed Lemna minor, and the results show that the nanoparticles are mainly accumulated in the epidermis of the fronds and roots [25]. A previous study [26] elucidated the uptake, translocation, and accumulation of synthesized iron oxide nanoparticles by pumpkin plants and showed that pumpkin cells can adsorb iron oxide particles. In the current experiment, the XRF spectroscopic analyses showed that the contents of CaO and Ca increased with increasing CaO levels. This is probably due to the CaO adhesion to the surface of the maize straw and subsequent degradation. The groups with 7% CaO concentration had the best adhesion in the current study, however the effect of moisture on ion attachment was not obvious. But the interaction between CaO and moisture had an extremely significant effect on CaO and Ca contents.

### Gas production from the maize straw treated with various level of CaO and moisture

Reducing the waste of straw is a significative issue which was explored by many scientists. We focused on whether utilization of maize straw could be improved in ruminants by of a change of its structure. Therefore, this study carried out research on *in vitro* gas production from straw in the rumen of ruminants. The *in vitro* experiment of the current study found that the maize straw treated with CaO increased gas production in the process of the fermentation. In the maize straw treated with 7% CaO the total gas production in 72 h was significantly higher than that of 0% and 3% groups, but there was no obvious difference from the 5% group. The gas produced in rumen mainly comes from the degradation of carbohydrate and carbon contents of protein in the feed by the activities of microorganisms [27]. *In vitro* gas production relates to the degradation activity of microorganisms and feed degradation rate in the rumen, and the amount of gas produced from the substrate reflects the degree that the feed is used by microorganisms. The degree of nutritional value of the substrate also influences the production of gas [28]. Chemical treatments alter cell wall composition and physical association between structural carbohydrates and lignin in straw, and thus the area of interaction for microorganisms is increased. The CaO treatment, changed the structure of maize straw, facilitating easy degradation by rumen microbes, increasing the digestibility in animals and increasing the gas production [11]. In the present study, the lignin content of the maize straws decreased significantly after CaO treatment and a previous study [29] also identified the higher degradation rate of lignin and digestibility of the maize straw following the treatment with CaO. Upward trend of gas production with increasing concentration of CaO treatment is consistent with several studies including in the current elucidation. The optimal treatment effect was seen in the group of 7% CaO. Rumen fermentation is directly influenced by the composition and the diversity of the rumen microorganisms [30], and the effect of CaO treatment on rumen microbial diversity and richness should be further explored.

## CONCLUSION

In the current study, maize straws were treated with various levels of CaO and with different moisture contents. Subsequently, these treated straws were comprehensively analyzed using XRF, SEM, and FTIR and gas production was investigated under anaerobic conditions in a simulated rumen environment. The test group with 7% CaO treatment was evaluated as the best treatment combination from the comprehensive analysis of XRF, SEM, and FTIR. The gas production increased with increasing levels of CaO (7%>5%>3%>0%). In summary, CaO treatment can adhere to the surface of the maize straw, and then improve the digestibility of the maize straw in ruminants by modifying the structure of lignocellulose and facilitating the maize straw for microbial degradation. Therefore, maize straw can be utilized by the feed industry.

## Figures and Tables

**Table 1:** Ingredients and chemical composition of the diets fed to dairy cattle

Total mixed ration	
Ingredient (g/kg DM)	
Corn meal	230
Wheat bran	42
Soya bean meal	52
Cottonseed meal	64
Dicalcium phosphate	4.2
NaCl	4.2
Premix^1)^	4.2
Alfalfa hay	449
Chinese wild rye hay	150
Chemical composition	
DM (g/kg)	527.5
NE_L_ (Mcal/kg)*	1.4
CP (g/kg DM)	158
aNDF (g/kg DM)	432
ADF (g/kg DM)	295

DM, dry matter; NE_L_, net energy for lactation; CP, crude protein; aNDF, neutral detergent fibre analysed with a heat-stable amylase; ADF, acid detergent fiber.

1) Contained 10% Mg; 5,000 mg/kg of Zn; 1,000 mg/kg of Mn; 2,500 mg/kg of Fe; 2,600 mg/kg of Cu; 192 mg/kg of I; 45 mg/kg of Co; 60 mg/kg of Se; 1,240,000 IU/kg of vitamin A; 50,000 IU/kg of vitamin D; 10,500 IU/kg of vitamin E.

* According to NRC (2001).

**Table 2:** The levels of various fiber components of maize straw treated with different concentrations of CaO and different moisture contents (%)

Ca	Moisture	NDF	ADF	ADL	Hemicellulose (%)	Cellulose (%)
0%	40%	75.85^a^	45.79^cd^	4.37^cde^	29.09^a^	40.97^abc^
	50%	77.33^a^	49.99^a^	5.32^abcd^	27.34^a^	43.38^a^
	60%	75.64^a^	50.23^a^	5.90^a^	25.84^ab^	42.40^ab^
3%	40%	70.68^b^	48.32^abcd^	5.51^abc^	22.35^bc^	41.19^abc^
	50%	70.78^b^	48.92^abc^	5.93^a^	21.12^cd^	42.28^ab^
	60%	71.05^b^	49.51^ab^	5.76^ab^	21.66^cd^	42.17^abc^
5%	40%	67.29^c^	46.97^abcd^	5.30^abcd^	20.46^cd^	40.30^abc^
	50%	65.44^de^	45.25^cd^	4.58^bcde^	20.59^cd^	38.97^c^
	60%	66.47^cd^	46.44^abcd^	4.69^abcde^	19.16^cd^	39.64^bc^
7%	40%	65.57^cde^	46.05^bcd^	4.75^abcde^	19.76^cd^	40.49^abc^
	50%	64.91^de^	44.86^d^	3.71^e^	19.24^cd^	40.04^abc^
	60%	64.42^e^	46.15^bcd^	4.26^de^	17.60^d^	40.59^abc^
SEM		0.645	1.312	0.444	1.397	1.482
Main effect						
CaO	0%	76.28^a^	48.67^a^	5.20^ab^	27.43^a^	42.25^a^
	3%	70.84^b^	48.91^a^	5.73^a^	21.71^b^	41.88^ab^
	5%	66.40^c^	46.22^b^	4.86^bc^	20.07^bc^	39.64^c^
	7%	64.97^d^	45.68^b^	4.24^c^	18.87^c^	40.37^bc^
Moisture	40%	69.85^a^	46.78^a^	4.98^a^	22.92^a^	40.74^a^
	50%	69.61^a^	47.25^a^	4.88^a^	22.07^a^	41.17^a^
	60%	69.40^a^	48.08^a^	5.15^a^	21.06^a^	41.20^a^
p-value	CaO	<0.01	<0.01	<0.01	<0.01	0.03
	Moisture	0.62	0.37	0.69	0.18	0.81
	CaO×moisture	0.20	0.33	0.15	0.95	0.80

NDF, neutral detergent fiber; ADF, acid detergent fiber; ADL, acid detergent lignin; SEM, standard error of mean.

a–e Values in the same column with different letter superscripts indicate that the samples have significant difference (p<0.05).

**Table 3:** The levels of secondary molecular structures of carbohydrate in the maize straw treated with various treatments (1×10^−3^)

Items		Lignin-H	Lignin-A	STCHO-H	STCHO-A	Cell-H	Cell-A	CHO-1-H	CHO-1-A	CHO-2-H	CHO-2-A	CHO-3-H	CHO-3-A
0%	40%	2.89^a^	104.51^a^	10.41^bcd^	1,644.17^ab^	9.47^a^	441.95^a^	7.71^a^	164.41^a^	5.11^ab^	108.05^abc^	44.44^b^	3,576.41^a^
	50%	2.90^a^	58.61^b^	11.06^bcd^	1,795.20^a^	8.94^ab^	444.28^a^	6.55^abcd^	136.77^abc^	4.84^ab^	113.99^ab^	103.30^a^	3,184.84^ab^
	60%	2.21^abcd^	44.99^b^	7.86^d^	1,362.24^ab^	7.08^abc^	354.82^a^	5.42^abcd^	110.03^bcde^	5.54^a^	82.54^abcd^	34.82^b^	2,791.18^abcd^
3%	40%	2.29^abc^	48.00^b^	11.72^abcd^	1,706.77^a^	6.69^bc^	332.64^ab^	6.32^abcd^	132.86^abcd^	3.48^ab^	74.81^abcd^	39.99^b^	3,112.08^abc^
	50%	1.38^cde^	25.01^b^	9.43^cd^	1,217.92^ab^	5.59^cd^	177.32^cd^	6.94^abc^	94.12^de^	4.29^ab^	107.16^abc^	30.52^b^	2,520.52^bcd^
	60%	1.58^cde^	27.94^b^	13.58^ab^	1,770.67^a^	4.15^de^	213.77^bc^	7.25^ab^	147.11^ab^	5.39^a^	122.27^a^	43.22^b^	3,328.47^ab^
5%	40%	1.23^e^	23.89^b^	8.75^cd^	1,084.78^b^	2.33^def^	109.97^cd^	4.55^cd^	94.31^de^	3.07^b^	58.74^d^	26.82^b^	2,134.71^cd^
	50%	1.67^bcde^	36.65^b^	10.40^bcd^	1,317.55^ab^	3.08^def^	156.87^cd^	4.88^bcd^	95.92^cde^	3.45^ab^	70.02^bcd^	31.36^b^	2,437.83^bcd^
	60%	1.46^cde^	27.16^b^	10.80^bcd^	1,407.40^ab^	2.14^def^	102.57^cd^	5.30^abcd^	111.3^5^^bcde^	3.76^ab^	86.30^abcd^	29.29^b^	2,468.34^bcd^
7%	40%	2.56^ab^	53.62^b^	15.25^a^	1,728.29^a^	3.71^def^	199.29^cd^	6.47^abcd^	131.28^abcd^	3.73^ab^	61.19^cd^	39.52^b^	3,115.63^abc^
	50%	1.31^de^	25.50^b^	9.40^cd^	1,098.69^b^	1.63^f^	74.30^d^	4.05^d^	82.10^e^	3.47^ab^	78.87^abcd^	25.43^b^	2,039.35^d^
	60%	1.85^bcde^	39.14^b^	12.68^abc^	1,406.63^ab^	1.74^ef^	88.48^cd^	4.71^cd^	93.06^de^	4.01^ab^	90.54^abcd^	30.44^b^	2,548.28^abcd^
SEM	0.34	15.96	1.48	0.32	0.90	47.60	0.90	14.66	0.81	17.08	19.65	369.79	
Main effect													
CaO	0%	2.67^a^	69.37^a^	9.78^b^	1,600.50^a^	8.50^a^	413.70^a^	6.56^a^	137.10^a^	5.17^a^	101.50^a^	60.85^a^	3,184.10^a^
	3%	1.75^b^	33.65^b^	11.58^ab^	1,565.10^a^	5.14^b^	241.20^b^	6.84^a^	124.70^ab^	4.39^ab^	101.40^a^	37.91^a^	2,987.00^ab^
	5%	1.45^b^	29.23^b^	9.98^b^	1,269.90^a^	2.52^c^	123.10^c^	4.91^b^	100.50^c^	3.43^b^	71.69^b^	29.16^a^	2,347.00^c^
	7%	1.91^b^	39.42^b^	12.44^a^	1,411.20^a^	2.36^c^	120.70^c^	5.08^b^	102.10^bc^	3.74^b^	76.87^ab^	31.79^a^	2,567.80^bc^
Moisture	40%	2.24^a^	57.51^a^	11.53^a^	1,541.0^a^	5.55^a^	271.00^a^	6.26^a^	130.70^a^	3.85^a^	75.70^a^	37.69^a^	2,984.70^a^
	50%	1.81^a^	36.44^ab^	11.23^a^	1,357.30^a^	4.56^ab^	213.20^ab^	5.67^a^	102.20^b^	4.02^a^	92.51^a^	47.65^a^	2,545.60^a^
	60%	1.78^a^	34.81^b^	10.07^a^	1,486.70^a^	3.78^b^	189.90^b^	5.60^a^	115.40^ab^	4.68^a^	95.41^a^	34.44^a^	2,784.10^a^
p-value	CaO	<0.01	0.01	0.09	0.2	<0.01	<0.01	0.02	<0.01	0.04	0.06	0.19	0.03
	Moisture	0.10	0.09	0.34	0.45	0.02	0.04	0.53	0.03	0.32	0.22	0.61	0.25
	CaO×moisture	0.15	0.46	0.04	0.19	0.59	0.35	0.36	0.04	0.97	0.47	0.36	0.36

Lignin-H, lignin height, centered at ca.1,531 to 1,486 cm^−1^; Lignin-A, lignin area, centered at ca. 1,531 to 1,486 cm^−1^; STCHO-H, structure carbohydrate height, centered at ca. 1,486 to 1,189 cm^−1^; STCHO-A, structure of carbohydrate area, centered at ca.1,486 to 1,189 cm^−1^; Cell-H, cellulose compound height, centered at ca.1,294 to 1,189 cm^−1^; Cell-A, cellulose compound area, centered at ca. 1,294 to 1,189 cm^−1^; CHO-1-H, total carbohydrate 1st height, centered at ca. 1,189 to 1,143 cm^−1^; CHO-1-A, total carbohydrate 1st area, centered at ca.1,189 to 1,143 cm^−1^; CHO-2-H, total carbohydrate 2nd height, centered at ca.1,143 to 1,087 cm^−1^; CHO-2-A, total carbohydrate 2nd area, centered at ca.1 143 to 1,087 cm^−1^; CHO-3-H, total carbohydrate 3rd height, centered at ca. 1,087 to 914 cm^−1^; CHO-3-A, total carbohydrate 3rd area, centered at ca. 1,087 to 914 cm^−1^; SEM, standard error of mean.

a–f Values in the same column with different letter superscripts indicate that the samples have significant difference (p<0.05).

**Table 4:** Correlation between secondary molecular structure and fiber components of maize straw from different treatment groups

Item	NDF		ADF		ADL		Hemicellulose		Cellulose	
				
	r	P	r	P	r	P	r	P	r	P
Lignin-H	0.62*	0.03	0.21	0.52	0.02	0.94	0.73**	<0.01	0.41	0.18
Lignin-A	0.57	0.06	−0.07	0.82	−0.18	0.57	0.76**	<0.01	0.18	0.57
STCHO-H	−0.27	0.39	−0.12	0.71	−0.13	0.69	−0.28	0.38	−0.04	0.89
STCHO-A	0.46	0.13	0.36	0.26	0.22	0.50	0.44	0.15	0.44	0.16
Cell-H	0.94**	<0.01	0.54	0.07	0.41	0.18	0.94**	<0.01	0.69*	0.01
Cell-A	0.92**	<0.01	0.52	0.08	0.36	0.25	0.94**	<0.01	0.65*	0.02
CHO-1-H	0.64*	0.02	0.44	0.15	0.45	0.14	0.60*	0.04	0.57	0.05
CHO-1-A	0.63*	0.03	0.29	0.36	0.21	0.51	0.67*	0.02	0.40	0.20
CHO-2-H	0.77**	<0.01	0.63*	0.03	0.42	0.18	0.68*	0.02	0.72**	<0.01
CHO-2-A	0.60*	0.04	0.49	0.10	0.27	0.40	0.46	0.13	0.66*	0.02
CHO-3-H	0.68*	0.02	0.52	0.08	0.24	0.46	0.64*	0.03	0.67*	0.02
CHO-3-A	0.65*	0.02	0.38	0.23	0.28	0.38	0.67*	0.02	0.51	0.09

NDF, neutral detergent fiber; ADF, acid detergent fiber; ADL, acid detergent lignin; r, correlation coefficient; Lignin-H, lignin height, centered at ca.1,531 to 1,486 cm^−1^; Lignin-A, lignin area, centered at ca. 1,531 to 1,486 cm^−1^; STCHO-H, structure carbohydrate height, centered at ca. 1,486 to 1,189 cm^−1^; STCHO-A, structure of carbohydrate area, centered at ca.1,486 to 1,189 cm^−1^; Cell-H, cellulose compound height, centered at ca.1,294 to 1,189 cm^−1^; Cell-A, cellulose compound area, centered at ca. 1,294 to 1,189 cm^−1^; CHO-1-H, total carbohydrate 1st height, centered at ca. 1,189 to 1,143 cm^−1^; CHO-1-A, total carbohydrate 1st area, centered at ca.1,189 to 1,143 cm^−1^; CHO-2-H, total carbohydrate 2nd height, centered at ca.1,143 to 1,087 cm^−1^; CHO-2-A, total carbohydrate 2nd area, centered at ca.1 143 to 1,087 cm^−1^; CHO-3-H, total carbohydrate 3rd height, centered at ca. 1,087 to 914 cm^−1^; CHO-3-A, total carbohydrate 3rd area, centered at ca. 1,087 to 914 cm^−1^.

* Means p<0.05,

** means p<0.01.

**Table 5:** The CaO and Ca contents in maize straw from different treatment combinations (%)

Treatments	Moisture	Contents	

		CaO (%)	Ca (%)
CaO			
0%	40%	22.73^f^	16.25^f^
	50%	20.29^g^	14.51^g^
	60%	20.02^g^	14.31^g^
3%	40%	56.32^e^	40.25^e^
	50%	56.29^e^	40.23^e^
	60%	56.39^e^	40.30^e^
5%	40%	62.51^d^	44.67^d^
	50%	62.76^d^	46.86^d^
	60%	64.46^c^	46.07^c^
7%	40%	71.19^a^	50.88^a^
	50%	71.00^a^	50.74^a^
	60%	69.45^b^	49.64^b^
SEM		0.517	0.370
Main effect			
CaO	0%	21.02^d^	15.02^d^
	3%	56.33^c^	40.26^c^
	5%	63.24^b^	45.20^b^
	7%	70.54^a^	50.42^a^
Moisture	40%	53.19^a^	38.01^a^
	50%	52.59^a^	37.58^a^
	60%	52.58^a^	37.58^a^
p-value	CaO	<0.01	<0.01
	Moisture	0.18	0.18
	CaO×moisture	<0.01	<0.01

SEM, standard error of mean.

a–g Values in the same column with different superscripts indicate that the samples have significant difference (p<0.05).

**Table 6:** Gas production from the variously treated maize straw at 6, 12, 24, 48, and 72 h in an *in vitro* rumen environment

Treatments		GP 6 (mL)	GP 12 (mL)	GP 24 (mL)	GP 48 (mL)	GP 72 (mL)
0%	40%	6.45^ab^	28.91^c^	47.68^bcd^	56.18^bcd^	65.30^c^
	50%	5.76^cd^	29.68^c^	45.01^d^	54.16^d^	68.10^c^
	60%	4.90^d^	28.75^c^	46.6^cd^	55.19^cd^	65.24^c^
3%	40%	6.71^ab^	30.90^bc^	44.17^d^	57.75^abcd^	70.64^abc^
	50%	6.57^ab^	32.49^abc^	45.98^cd^	55.56^cd^	66.61^c^
	60%	5.84^cd^	29.47^c^	44.03^d^	54.84^cd^	69.79^abc^
5%	40%	6.59^ab^	31.99^bc^	46.84^cd^	56.46^bcd^	66.81^c^
	50%	8.17^a^	32.15^abc^	51.49^ab^	62.01^ab^	71.96^abc^
	60%	7.43^ab^	29.16^c^	46.99^cd^	60.66^abc^	75.11^ab^
7%	40%	7.76^ab^	33.37^abc^	49.54^abc^	60.09^abcd^	69.56^bc^
	50%	8.78^a^	34.98^ab^	51.32^ab^	62.90^a^	76.43^a^
	60%	8.80^a^	36.96^a^	52.91^a^	60.36^abc^	75.98^ab^
SEM		0.492	1.764	1.356	2.123	2.200
Main effect						
CaO	0%	5.70^c^	29.11^b^	46.43^bc^	55.18^b^	66.21^c^
	3%	6.37^c^	30.95^b^	44.72^c^	56.04^b^	69.01^bc^
	5%	7.40^b^	31.10^b^	48.44^b^	59.71^a^	71.30^ab^
	7%	8.44^a^	35.10^a^	51.26^a^	61.12^a^	73.99^a^
Moisture	40%	6.88^a^	31.29^a^	47.06^a^	57.62^a^	68.08^b^
	50%	7.32^a^	32.33^a^	48.45^a^	58.66^a^	70.77^ab^
	60%	6.74^a^	31.09^a^	47.63^a^	57.76^a^	71.53^a^
p-value	CaO	<0.01	<0.01	<0.01	<0.01	<0.01
	Moisture	0.23	0.57	0.35	0.76	0.11
	CaO×moisture	0.08	0.63	0.08	0.47	0.19

GP6, total gas production within 6 h; GP12, total gas production within 12 h; GP24, total gas production within 24 h; GP48, total gas production within 48 h; GP72, total gas production within 72 h; SEM, standard error of mean.

a–d Values in the same column with different superscripts indicate that the samples have significant difference (p<0.05).
